# Characterization of Andean Blueberry in Bioactive Compounds, Evaluation of Biological Properties, and In Vitro Bioaccessibility

**DOI:** 10.3390/foods9101483

**Published:** 2020-10-17

**Authors:** Nieves Baenas, Jenny Ruales, Diego A. Moreno, Daniel Alejandro Barrio, Carla M. Stinco, Gabriela Martínez-Cifuentes, Antonio J. Meléndez-Martínez, Almudena García-Ruiz

**Affiliations:** 1 Department of Food Technology, Food Science and Nutrition, Faculty of Veterinary Sciences, Regional Campus of International Excellence “Campus Mare-Nostrum”, University of Murcia, Campus de Espinardo, 30100 Murcia, Spain; 2 Department of Food Science and Biotechnology, Escuela Politécnica National, Quito 17-01-2759, Ecuador; jenny.ruales@epn.edu.ec (J.R.); ybagcmarti@hotmail.com (G.M.-C.); almudena.garcia@imdea.org (A.G.-R.); 3 Phytochemistry and Healthy Foods Lab., Department of Food Science and Technology, CEBAS-CSIC, Campus de Espinardo-Edificio 25, E-30100 Murcia, Spain; dmoreno@cebas.csic.es; 4 Universidad Nacional de Río Negro, CIT Río Negro, Don Bosco y Leloir s/n, 8500 Río Negro, 8500 Viedma, Argentina; drbarrio@unrn.edu.ar; 5 Food Colour & Quality Lab., Department of Nutrition & Food Science, Universidad de Sevilla, Facultad de Farmacia, 41012 Sevilla, Spain; cstinco@us.es (C.M.S.); ajmelendez@us.es (A.J.M.-M.); 6 Laboratory of Epigenetics of Lipid Metabolism, Madrid Institute for Advanced Studies (IMDEA)-Food, CEI UAM + CSIC, 28049 Madrid, Spain

**Keywords:** mortiño, *Vaccinium floribundum*, HPLC–MS/MS, anthocyanins, antioxidant, antimicrobial, toxicity, zebrafish

## Abstract

The aim of this study was to evaluate Andean blueberries (*Vaccinium floribundum* Kunth) from Ecuador as a potential functional ingredient for the food and pharmaceutical industries. The analysis of bioactive compounds by HPLC–DAD–MS^n^ determined a high content of (poly)phenols, mainly anthocyanins, and the presence of the carotenoid lutein. Regarding its biological properties, Andean blueberry did not show toxicity by the zebrafish embryogenesis test, showing also a lack of the antinutrients lectins. Moreover, the results of in vitro and in vivo antioxidant capacity evaluation suggested its possibility to be used as natural antioxidant. This fruit also exhibited antimicrobial activity toward *Staphylococcus aureus* and *Escherichia coli* in low doses. Finally, in vitro gastrointestinal (GI) digestion showed a partial bioaccessibility of (poly) phenols (~50% at the final step), showing high antioxidant capacity in the different GI phases. These results revealed Andean blueberry as an interesting candidate for being used as a functional ingredient and the development of further in vivo and clinical assays.

## 1. Introduction

The consumption of berries has been associated with health-promoting effects, such as reductions in the incidence of degenerative and chronic diseases (cardiovascular diseases, type 2 diabetes, and certain types of cancer, among others), mainly due to the presence of bioactive compounds (phenolic compounds, vitamins, and carotenoids), associated with radical scavenging capacity and epigenetic mechanisms [1]. Clinical intervention studies have also shown that phenolic compounds from berries, particularly anthocyanins, are able to improve the profile of inflammatory markers and the total antioxidant status, these effects being more evident with chronic dietary interventions [2].

Andean blueberry (*Vaccinium floribundum* Kunth), also known as mortiño, is a promising wild berry of the family Ericaceae that grows spontaneously in the Andean regions of Ecuador. The demand for these small (~8 mm diameter), black, and round fruits has been increasing due to their antioxidant characteristic, similar to other *Vaccinium* species, such as cranberry, blueberry, or bilberry, mostly related to the high content of (poly) phenolic compounds.

The phytochemical evaluation of these fruits is essential to assess their potential health-promoting effects before an intervention study, establishing their characteristics for use in the food, nutraceutical, and pharmaceutical industries. Unlike many other Ibero-American fruits and vegetables, the carotenoid profile of Andean blueberry is basically unknown [3]. The study of carotenoids is very important as they are very versatile compounds with many applications in agro-food and nutricosmetics [4,5]. As far as we know, only few works have published the profile and content of phenolic compounds in *V. floribundum* Kunth evaluated by HPLC–MS/MS [6,7]. These results are varied and influenced by many factors, including differences among varieties, maturity of the fruit, environmental parameters, and pre-/postharvest handling [8]. Aside from phytochemical evaluation, in vitro antioxidant capacities [7,9] and antimicrobial activities [10] have been reported in Andean blueberry. However, further experiments are required before taking a step ahead through in vivo assays and clinical trials. In this sense, the aim of this study was to evaluate the phytochemical profile of *V. floribundum* Kunth from the local market in Machachi (Ecuador) by HPLC–DAD–MS/MS and assess its antioxidant capacity in vitro by ABTS^·^ (2,2′-Azino-bis(3-ethylbenzothiazoline-6-sulfonic acid) diammonium salt), DPPH^−^ (2,2-Diphenyl-1-picrylhydrazyl) and ORAC (oxygen radical absorbance capacity) methods and its antimicrobial activity against *Staphylococcus aureus* and *Escherichia coli*, identifying substantial differences with previous reports. In addition, the in vivo toxicity effect by the zebrafish embryogenesis test and the in vivo antioxidant capacity using the zebrafish animal model (thiobarbituric acid reactive substances (TBARS) test) were evaluated for the first time, simulating physiological conditions through an aqueous extract. Additionally, the presence of lectins in Andean blueberry as antinutritional factors were newly investigated. Finally, the bioaccessibility of phenolic compounds was studied after an in vitro gastrointestinal digestion, evaluating also the antioxidant activity in the different phases of digestion, which may ultimate the physiological effect and role of Andean blueberry within the organism. These results make advances in the knowledge about the health benefits linked to Andean blueberry consumption related to bioactivity, bioaccessibility, and safety, being essential before carrying out further in vivo assays and clinical trials.

## 2. Materials and Methods

### 2.1. Fruit Samples

Andean blueberry (*Vaccinium floribundum* Kunth) was purchased at a local market in Machachi, Ecuador. These fruits were selected, washed with tap water, disinfected using 100 ppm of chlorine, and freeze-dried. Then, samples were packed and stored at −20 °C pending analysis.

### 2.2. Standards, Chemicals, and Solvents

The standards 5-*O*-caffeoylquinic acid and rutin (quercetin-3-rutinoside) were acquired from Sigma-Aldrich Chemie GmbH (Steinheim, Germany). Cyanidin 3-*O*-glucoside was obtained from Polyphenols Laboratories (Sandnes, Norway). Lutein was obtained according to the method described by Meléndez-Martínez et al. [11]. Trolox (6-hydroxy-2,5,7,8-tetramethylchroman-2-carboxylic acid) was purchased from Fluka Chemika (Neu-Ulm, Switzerland). The reagents 2,2-diphenyl-1-picrylhydrazyl radical (DPPH^∙^), 2,2-azino-bis(3-ethylbenzothiazoline-6-sulfonic acid) diammonium salt (ABTS^∙+^), monobasic and dibasic sodium phosphate, Folin–Ciocalteu reagent, and fluorescein (free acid) were purchased from Sigma-Aldrich Chemie GmbH (Steinheim, Germany). Solvents of analytical grade ethanol, methanol, hexane, acetone, dichloromethane, acetonitrile, and formic acid were obtained from Merck (Darmstadt, Germany).

### 2.3. Physicochemical Analysis

Weight, length, and diameter were determined according to the methods described by the Ecuadorian Technical Standards 2427 [12]. The pH of the fruits was analyzed using a pH meter, SevenCompact pH/Ion S220 (Mettler Toledo, Greifensee, Switzerland), following the method described by the Association of Official Analytical Chemists (AOAC method 981.12) [13]. The method used to determine the moisture of the fruits included drying to constant weight in a vacuum oven at 70 °C and 100 mmHg pressure until a high level of water evaporation (max. 6 h) (AOAC method 950.27) was reached [13]. The results were expressed in percentage (g H_2_O 100 g^−1^ of sample). Soluble solids were assayed using a Portable Brix Refractometer VBR90S (Boeco, Germany) (AOAC method 931.12) [13]. The results were expressed as °Brix. The titratable acidity was determined by the method suggested by AOAC (method 942.15) [13], performing titration with NaOH 0.1 N. The results were expressed in % of citric acid. Every assay was conducted in triplicate.

### 2.4. Identification and Quantification of Phenolic Compounds by HPLC–DAD–ESI/MS^n^

The analysis of phenolic compounds was carried out following the protocol and method of Gironés-Vilaplana et al. [14]. Briefly, 100 mg of the sample was extracted with 1 mL of methanol/water/formic acid (70:29:1, *v*/*v*/*v*), mixed in a vortex for 60 s, kept in an ultrasonic bath for 60 min, and then maintained at 4 °C overnight and sonicated again for 60 min. The efficiency of the extraction was confirmed by a second extraction of the pellet, showing that more than 95% of (poly) phenols were obtained in the first stage. Hereafter, the samples were filtered (0.22 μm PVDF filter, Millex HV13, Millipore, Bedford, MA, USA), and the identification of phenolic compounds was carried out following their MS and MS^2^ fragmentation ions by HPLC–DAD–ESI/MS^n^, composed of an HPLC Agilent 1100 coupled to a mass spectrometer detector (model G2445A) equipped with an electrospray ionization interface (Agilent Technologies, Waldbronn, Germany). Ionization conditions were selected according to the method, covering a full-scan mass range from *m*/*z* 100 to 1200. The mass spectrometry data (MS^n^) analysis was performed in the negative ionization mode for flavonols and phenolic acids, except for anthocyanins, where the positive ionization mode was used. The quantification of phenolic compounds was performed in an HPLC–DAD Agilent 1220 Infinity system equipped with a Luna C18 column (25 cm × 4.6 mm, 5 μm particle size; Phenomenex, Macclesfield, UK) using the acquisition conditions described in the method. Phenolic acids were quantified using as external standard 5-*O*-caffeoylquinic acid at 320 nm, flavonols at 360 nm using the standard rutin (quercetin-3-rutinoside), and anthocyanins by using the standard cyanidin 3-*O*-glucoside at 520 nm [14]. The samples were extracted and analyzed in triplicate. Results were expressed as µg g^−1^ dry weight (DW).

### 2.5. Identification and Quantification of Isoprenoids (Carotenoids and α-Tocopherol) by RRLC

The extraction and quantification of carotenoids were conducted according to the method described by Stinco et al. [15]. Briefly, the samples (200 mg) were extracted with 1 mL of hexane/acetone (1:1 *v*/*v*) using a vortex and an ultrasonic bath for 2 min. Next, the samples were centrifuged (18,000× *g* for 5 min), and the colored fractions were recovered. The extraction was repeated twice more up to pellet color extinction. Ultimately, the carotenoid-combined extracts were dried in a rotary evaporator at temperature below 30 °C. To obtain saponified carotenoids, the extracts were treated with 1 mL of dichloromethane and 1 mL of methanolic KOH (30% *w*/*v*) for 1 h under dim light at room temperature Then, the samples were washed with water to remove any trace of base. The extracts obtained were dried in a rotary evaporator and redissolved in ethyl acetate prior to their analysis in the RRLC system. The samples were extracted and analyzed in triplicate. The RRLC acquisitions were made by using an Agilent 1260 system equipped with a diode array detector, scanning from 200 to 770 nm, and a Poroshell 120 C18 column (2.7 µm, 5 cm × 4.6 mm) (Agilent, Palo Alto, CA) set at 28 °C. Chromatograms were recorded at 450 nm. The identification and quantification of isoprenoids were performed by chromatographic UV–VIS spectroscopic characteristics and retention time comparison with the standards, as well as by comparison with the external calibration line calculated [15]. Results were expressed as µg/g DW.

### 2.6. Zebrafish Larvae Collection and Toxicity Test

Adult male and female wild-type zebrafish (*Danio rerio*) were obtained from a commercial fish farm, and 10 fish were kept in a 10 L glass tank under the following conditions: 28.5 °C, with a 16/8 h light–dark cycle. The fish were fed 3 times a day, 6 days a week, with TetraMin flake food supplemented with live brine shrimps (*Artemia salina*). The zebrafish embryo toxicity test was performed using the method of Murphey and Zon [16], with some modifications. Embryos were obtained by photoinduced spawning over green plants and then cultured at 28 °C in a fishbowl. Five-hour post fecundation larvae (30 larvae per well) were incubated in 24-well plates with 1 mL of water. Freeze-dried Andean blueberry was extracted in water under constant agitation for 30 min; then samples were centrifuged (10,000× *g*, 5 min), and the supernatant was filtered. Andean blueberry was added at different concentrations (10.0, 5.0, 2.0, 1.0, 0.5, 0.2, and 0.1 mg/mL) in 200 μL of water. The effect of the extract on the larvae was measured after 48 h. At the end of the incubation time, larvae mortality and morphologic changes were observed under microscope, determining the percentage of dead larvae. In the control wells, there should be less than 10% of dead eggs (coagulation of fertilized eggs, lack of somite formation, lack of tail detachment from the yolk sac, and lack of heartbeat).

### 2.7. Antioxidant Capacity

#### 2.7.1. Antioxidant Capacity In Vitro

The antioxidant capacity was evaluated by the methods DPPH^−^, ABTS^·^, and ORAC adapted to a microscale and using 96-well microplates (Nunc, Roskilde, Denmark), which were measured using an Infinite^®^ M200 microplate reader (Tecan, Grödig, Austria). The power of scavenge DPPH^−^ and ABTS^∙^ radicals was determined according to the method described by Mena et al. [17]. Briefly, 2 µL of the corresponding diluted sample (previously extracted for phenolic compound analysis) was added to the wells containing the stock solution (250 µL) with absorbance ~1. Then, the plate was shaken and left for 50 min at 37 °C; thus the variation in absorbance was measured at 515 or 414 nm for the DPPH^−^ or ABTS^∙^ methods, respectively. Regarding the ORAC method [18], 25 µL of the properly diluted sample was added to 150 µL of fluorescein (1 µM), and after 30 min of incubation, 25 µL of the radical AAPH (2,2′-azobis(2-methylpropionamidine)-dihydrochloride) (250 mM) was added to the wells. Results were evaluated by measuring the variation in fluorescence every 2 min during 120 min of reaction with the AAPH radical. Trolox was used as standard in all the methods, following the same procedure as with the samples. According to the solubilizing agents used in the methods, the samples were dissolved in MeOH in the DPPH^−^ method to evaluate less polar compounds, while in the ABTS^·^ and ORAC methods, the samples were dissolved in water to evaluate more polar compounds. Results were expressed as µmol Trolox equivalents (TE) g^−1^ DW. Assays were carried out in triplicate.

#### 2.7.2. Antioxidant Capacity In Vitro: Thiobarbituric Acid Reactive Substances (TBARS) in Zebrafish Larvae Model

The TBARS method in zebrafish larvae was used as described by Carrillo et al. [19]. Briefly, freeze-dried Andean blueberry was extracted in water under constant agitation for 30 min; then samples were centrifuged (10,000× *g*, 5 min), and the supernatant was filtered. Five days post fecundation, larvae were incubated in 24-well plates (30 larvae/well in triplicate) with different concentrations of Andean blueberry (1.0, 0.5, 0.2, and 0.1 mg/mL). Groups of 30 larvae/well in aquarium water were used as negative control. Lipid peroxidation was started by adding 1 mL of 500 μM H_2_O_2_ and incubating for 8 h at 28 °C. Next, H_2_O_2_ was removed, and 500 μL of 0.1% Tween was added. Larvae were mixed and homogenized using an Ultra-Turrax (T25 basic Ultra-Turrax IKA, Thermo Fisher Scientific, Karlsruhe, Germany). After that, 1 mL of 1% TBA (thiobarbituric acid) was added, and the solution was heated up to 95 °C for 1 h, and then cooled for 15 min. Absorbance of the final solution was measured at 532 nm using a spectrophotometer (Evolution 200, Thermo Scientific, Karlsruhe, Germany). The synthetic antioxidant butylated hydroxytoluene (BHT) was used as positive control (0.1 mg/mL). The values of antioxidant capacity were expressed as the percentage of inhibition of lipid peroxidation in larvae homogenate as follows:

% inhibition of lipid peroxidation = ((Ab − As)/Ab) × 100(1)

where Ab is the absorbance of blank and As is the absorbance of the sample.

### 2.8. Determination of Antinutritional Factor: Lectins

The lectin content was determined through a hemagglutination assay following the protocol of Boeri et al. [20]. Briefly, twofold serial dilutions of Andean blueberry (2.5, 1.25, 0.625, 0.312, and 0.156 mg mL^−1^) in 10 mM phosphate-buffered saline (PBS, pH 7.4, 50 μL) were mixed with 50 μL of 4% human erythrocyte (group 0, Rh+) suspension in 96-well microtiter plates at 30 °C. The results of agglutination were visible macroscopically in the plate wells, observed after 1 h of incubation.

### 2.9. Antimicrobial Activity

Bacterial strains *Escherichia coli* ATCC 25922 and *Staphylococcus aureus* ATCC 25923 were used for the screening of antibacterial activity. The antibacterial assays were performed using the method of García-Ruiz et al. [21], with some slight modifications. Inhibition of microbial growth by Andean blueberry was determined by the microtiter dilution method using serial double dilutions of the antimicrobial agent (from 10 to 0.078 mg mL^−1^) and initial inoculum of 5 × 10^5^ CFU mL^−1^. Bacterial growth was determined by reading the absorbance at 620 nm. Growth inhibitory activity was expressed as a mean percentage of growth inhibition with respect to a control without antimicrobial sample as follow:

% inhibition of bacterial growth = ((Ab − As)/Ab) × 100(2)

where Ab is the absorbance of blank and As is the absorbance of the sample.

### 2.10. In Vitro Gastrointestinal Digestion

An in vitro gastrointestinal digestion procedure mimicking the physiological situation in the oro-gastrointestinal transit was used according to the method of Villacis-Chiriboga et al. [22], with modifications. Briefly, 0.5 g of free-dried Andean blueberry was dissolved in 11 mL of ethanol−water (6:94), was adjusted to pH 7 with 3 mL of artificial saliva (0.22 g/L CaCl_2_, 6.2 g/L NaCl, 2.2 g/L KCl, 1.2 g/L NaHCO_3_), and was shaken (150 rpm) for 2 min at 37 °C (oral phase). Then, the samples were adjusted to pH 2.0 with HCl 5 M, and subjected to incubation in a water bath (Precision Scientific model 25, Chicago, IL, USA) at 37 °C for 2 h under constant stirring of 150 rpm in the presence of 0.7 mL of stomach solution (20 mg of porcine pepsin and 0.7 mL of 0.1 M HCl) (gastric phase). The gastric digests were maintained in ice for 10 min to stop pepsin digestion. After this, the samples were adjusted to pH 6.0 with NaHCO_3_ (20 g 100 mL^−1^), and 2.25 mL of intestinal solution (18 mg of pancreatin with 112.5 mg of bile salts was dissolved in 4.5 mL of NaHCO_3_ 1 N) (intestinal initial phase). To stop intestinal digestion, the sample was kept for 10 min in an ice bath. Then, the sample was adjusted to pH 7.5 with NaHCO_3_ 1 N at 37 °C for 2 h under constant stirring of 150 rpm (intestinal final phase). Finally, the sample was adjusted to pH 7.2 with NaOH 0.5 M and centrifuged at 5000 rpm for 20 min at 4 °C (digestion final phase). Aliquots of 2 mL of digested sample in each phase were transferred to 2.0 mL Eppendorf tubes and centrifuged at 5000 rpm for 20 min at 4 °C in a Centrifuge 5415 D (Eppendorf, Hamburg, Germany). Supernatants obtained were used to determine the antioxidant capacity by the ABTS^·^ method (described in Section 2.7.1.), expressed as µmol TE g^−1^ DW, and phenolic contents (bioaccessible fraction), expressed as mg of gallic acid equivalents (GAE) per gram DW. Bioaccessibility (%) was calculated as the percentage of total phenolic compound content remaining in the bioaccessible fraction related to the original nondigested sample.

bioaccessibility% = 100 × (bioaccessible phenol content/TPC)(3)

### 2.11. Total Phenolic Content

Total phenolic content (TPC) of Andean blueberry samples was determined colorimetrically using the Folin–Ciocalteu reagent as described by Slinkard and Singleton [23]. Briefly, 500 µL of the extracts, blank or standards, were placed in a 15 mL tube, where 2.5 mL of the Folin–Ciocalteu reagent was added, allowing for reaction for 2 min while shaking. Then, 2 mL of a solution of sodium carbonate (75 g/L) was added and properly mixed. The solution was thus incubated 15 min at 50 °C. After that, the absorbance was measured at 750 nm in a spectrophotometer (Shimadzu UV-160A, Kyoto, Japan). Gallic acid was used as standard (10–90 mg/L), and the results were expressed as mg GAE g^−1^ DW. This assay was carried out in triplicate.

### 2.12. Statistical Analysis

All analyses were conducted in triplicate, mean values (*n* = 3) ± standard deviation (SD). The data were processed using GraphPad Prism 6 (La Jolla, CA, USA). A multifactorial analysis of variance (ANOVA) and Tukey’s multiple-range test were carried out to determine significant differences at *p*-values < 0.05. A nonlinear regression “dose-response inhibition” was used to determine the IC_50_ in zebrafish experiments.

## 3. Results and Discussion

### 3.1. Physicochemical Characterization

Andean blueberry fruits (*Vaccinium floribundum* Kunth) had high water content (~89%) and appropriate size (weight, length, and diameter) within the quality standards for blueberries (Table 1); nevertheless, these parameters are very varied among species and varieties [24,25]. Sugar concentration and pH are important parameters for evaluating blueberry quality. This fruit had low pH (2.6), titratable acidity (TTA) of 1.6%, and high amount of soluble sugars (11.2 °Brix), according to the expected range of pH 2.7–3.8, TTA values between 0.3% and 1.3%, and >11 °Brix reported for other blueberry cultivars, these values also being influenced by environmental and growing conditions [26,27,28].

In this sense, Andean blueberry is a sweet fruit with a pleasant acid flavor that could be consumed not only fresh but also as derived products, such as juice, jam, jelly, or wine, or could be used as food ingredient with potential technological applications, such as antioxidant and dying properties [26,29].

### 3.2. Identification and Quantification of Bioactive Compounds

In Andean blueberry fruits, mainly phenolic compounds were detected and one carotenoid was found. The characterization of phenolic compounds of these fruits was performed by the identification of individual compounds by HPLC-DAD-ESI/MS^n^ (Table 2) and the subsequent quantification using HPLC-DAD (Table 3), revealing a wide range of different (poly) phenols. A total of 16 phenolic compounds were identified following their main ion [M−H]^−^ (*m*/*z*) and MS^n^ fragmentation ions.

Four hydroxycinnamic acids were found, all of them being caffeoyl acid derivatives. Compound **1** was found as an adduct of 3-*O*-caffeoylquinic acid; this dimer is usually formed as an artefact of the mass spectrometry analysis, having a [2M−H]^−^ adduct ion at *m*/*z* 707 and [M−H]^−^ ion at *m*/*z* 353, which produced MS^2^ ions at *m*/*z* 191 and 179, which evidenced its tentative identification [30]. The 5-*O*-caffeoylquinic acid (**2**) also showed [M−H]^−^ ion at *m*/*z* 353, and the daughter ion at *m*/*z* 191. Compound **9**, caffeoylshikimic acid, gave its characteristic [M−H]^−^ ion at *m*/*z* 335 with MS^2^ fragmentation peaks at *m*/*z* 179, 161, and 131 [6,7,31]. Finally, compound **10** exhibited [M−H]^−^ ion at *m*/*z* 433 and gave MS^2^ fragmentation peaks at *m*/*z* 323, 179, and 161, being characteristic of caffeoylquinic acid derivatives [32]. This information, along with its characteristic spectrum with absorption at 320 nm, led us to the tentative identification of this compound as caffeic acid derivative, according to previous works analyzing *V. floribundum* [6] and *Vaccinium meridionale* [33].

Compounds **3**–**8** were detected as glycosylated anthocyanin derivatives of delphinidin and cyanidin, with the typical molecular ion at *m*/*z* 303 and 287, respectively, bound to a glucose or pentose, with a loss of 162 or 132 mass units, respectively. This anthocyanin profile agrees with previous works analyzing Andean blueberry [6,7]. Compounds **11–16** belonged to the flavonoid family, all of them being derivatives of quercetin, with the typical MS^2^ fragment of *m*/*z* 301 and a loss of 162 mass units in case of glucose, 132 due to pentose, and 146 because of the deoxyhexoside rhamnose. Other authors also found quercetin-3-glycosides as the predominant flavonols in this fruit [3]. Additionally, small amounts of two different myricetin derivatives were identified in mortiño berries [4].

The quantification of phenolic compounds (Table 3) showed anthocyanins as the main group present in the samples (~60% of the total phenolic compounds). Among them, cyanidin-3-*O*-pentoside and cyanidin-3-*O*-hexoside I were the predominant anthocyanins (~80% of the total), followed by delphinidin hexosides, accounting for 19%. These results agree with the distribution of anthocyanins described in *V. floribundum* before, showing anthocyanin contents in the range 3–10 mg/g DW, mainly constituted by cyanidin glycosides [6,7,34]. This accumulation of delphinidin and cyanidin-type anthocyanins has been related to the deep purple-black color of berries, these contents being affected by differences in the growth conditions or ripening stage of the fruits [35].

Regarding flavonols, these compounds accounted for 24% of the total phenolic compounds, all of them being quercetin glycosides. The contents of quercetin-3-*O*-hexoside I and quercetin-3-*O-*pentoside III were significantly high, corresponding to 70% of the total flavonols, as reported by You et al. [36].

Finally, hydroxycinnamic acids constituted 15.7% of the total, mainly represented by caffeoylquinic acids, the isomer of the chlorogenic acid 5-*O*-caffeoylquinic acid being the most representative compound (Table 3), according to previous studies showing chlorogenic acid derivatives as the main phenolic acids in *V. floribundum* [6,33].

Diverse contents of phenolic acids (1–3 mg g^−1^ DW) and flavonols (2–4 mg g^−1^ DW) were described before by HPLC in Andean blueberry [6,7,33,36], as several factors may affect the concentration of total phenolic compounds in blueberries, such as agronomic factors, cultivars and varieties, geographic region, storage conditions, ripeness, climate, and others, which are reported in the literature with varied contents of total phenolic compounds in *Vaccinium* sp. (0.5–7 mg g^−1^ FW; ~5–40 mg g^−1^ DW) [6,33,36].

On the other hand, the carotenoid content was studied using a rapid resolution liquid chromatography (RRLC) by comparing the chromatographic UV–VIS spectroscopic characteristics with the standards. Results showed lutein (5.94 µg g^−1^ DW = 0.67 µg g^−1^ FW) as the only carotenoid found in Andean blueberry (Table 3). Recently, other authors showed lutein as the main carotenoid in higher concentrations (8.7 µg g^−1^ FW), but also β-carotene in lower amounts (0.7 µg g^−1^ FW) [9]. On the other hand, only β-carotene (0.4 µg g^−1^ FW) was found in Andean blueberry by Vasco et al. [7]. These differences among Andean blueberry fruits affirm that similar varieties may contain diverse individual and total bioactive compounds depending on factors of different nature, including stage of maturity, variety, harvesting season or production, postharvest processing, and storage conditions, among others [37].

### 3.3. Embryo Toxicity Test with Zebrafish

**Figure 1:**
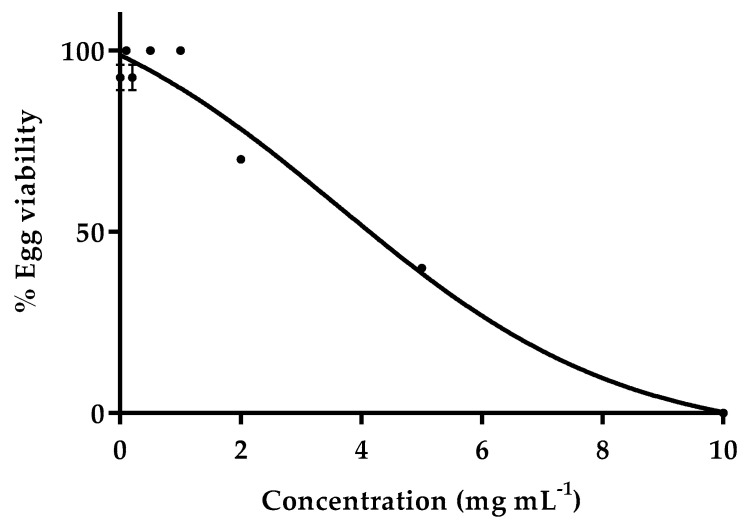
The embryotoxic effect expressed as % egg viability of various concentrations of Andean blueberry in the range 0.1–10 mg mL^−1^. Aquarium water was used for 100% egg viability. Results are mean values (*n* = 3) ± standard deviation.

Testing toxicity in preclinical studies comprises the evaluation of physiological, biological, or molecular alterations by in vitro or in vivo models. In this work, the toxic effect of Andean blueberry was evaluated by the zebrafish embryo toxicity test, determining the percentage of egg viability after 48 h of incubation with seven different concentrations of Andean blueberry extract (0.1, 0.2, 0.5, 1, 2, 5, and 10 mg mL^−1^). Results showed a 100% egg viability up to a concentration of 1 mg mL^−1^, with no morphological abnormalities found in the growth of the body, being that the toxicity of this aqueous extract strongly increased with higher concentrations (Figure 1).

Furthermore, we determined the LC_50_ dose of Andean blueberry that was able to cause 50% death of test animals in 48 h, LC_50_ = 3.63 mg mL^−1^. According to the distinct toxicity categories of aqueous substances against zebrafish, Andean blueberry extract could be included in the safe category, as harmful concentrations are considered up to 100 mg L^−1^ [38,39]. Based on the results, the maximum concentration causing no mortality (1 mg mL^−1^) was selected to evaluate the antioxidant capacity in vivo in a further experiment with zebrafish (Section 3.5).

### 3.4. Antioxidant Capacity In Vitro

Since there are multiple mechanisms involved in the oxidative stress in the human body, there is no universal method by which antioxidant capacity can be assessed accurately and quantitatively [40]. For in vitro analysis, it is recommended that at least two methods be used to provide reliable results regarding antioxidant capacity [41]. Thus, in this work, the DPPH^−^ and ABTS^·^ methods were selected to assess the relative antioxidant capacity for scavenging radicals, as DPPH^−^ radicals were dissolved in MeOH while ABTS^·^ cations were dissolved in water, examining both hydrophilic and lipophilic antioxidants in Andean blueberry. Besides, the oxygen radical absorbance capacity (ORAC) method was used to evaluate the capacity for scavenging free radicals by competition with the reference free radical scavenger (fluorescein) for the peroxyl radical (AAPH) in hydrophilic medium, which reflects physiological relevant perturbations [42]. Results showed higher antioxidant capacity values with the methods ABTS^·^ and ORAC (278 and 402 µmol TE g^−1^ DW, respectively) compared with the DPPH^−^ assay, which reported the lowest value (85 µmol TE g^−1^ DW) (Table 4). This fact can be explained by the high polarity of anthocyanins, the main phenolic group found in berries, which better contribute to the antioxidant activity in hydrophilic media. On the other hand, differences among these antioxidant capacity methods have been reported before, showing that DPPH^−^ is more selective than the ABTS^·^ and ORAC methods in the reaction with hydrogen atom donors, not reacting, for example, with OH groups from aromatic compounds [43].

Previous works have reported ORAC values of blueberries in the range 350–1000 µmol TE g^−1^ DW [44,45], ABTS^·^ results from 100 to 300 µmol TE g^−1^ DW [10,26], and DPPH^−^ values of 70–350 µmol TE g^−1^ DW [46], reflecting great differences among genotypes, varieties, development and ripening stage of the fruit, and contents of (poly) phenolic compounds. Thus, Andean blueberry can provide a good source of antioxidants in the diet, with potential benefits to human health.

### 3.5. Antioxidant Capacity In Vitro

**Figure 2:**
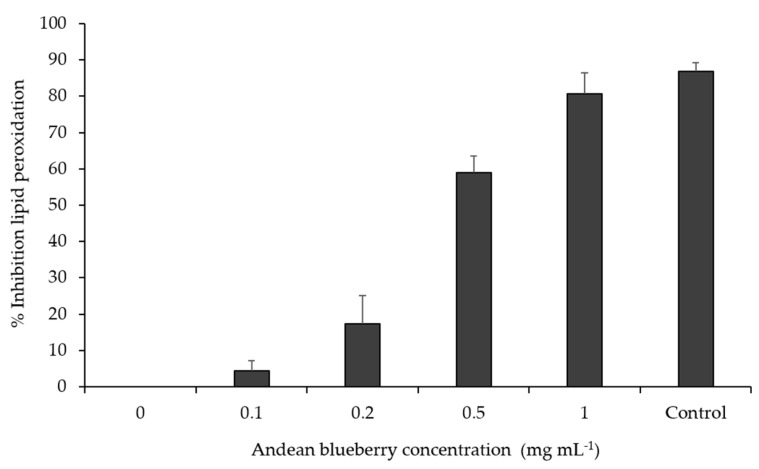
Inhibition of the lipid peroxidation in vivo using the zebrafish model. The zebrafish embryos were treated with concentrations of Andean blueberry in the range 0.1–1 mg mL^−1^. Butylated hydroxytoluene (BHT) was used as positive control (0.1 mg/mL). Results are mean values (*n* = 3) ± standard deviation.

The in vivo antioxidant activity was studied by the thiobarbituric acid reactive substances (TBARS) test using zebrafish larvae. In this experiment, the potential capacity of freeze-dried Andean blueberry to inhibit lipid peroxidation was assessed. In this work, doses from 1 to 0.1 mg mL^−1^ of Andean blueberry extracts were used to determine the antioxidant capacity in vivo, using as the maximum concentration the one previously selected in the toxicity test causing no mortality (1 mg mL^−1^). The concentration of Andean blueberry that was able to inhibit 50 % of the lipid peroxidation was 0.437 mg mL^−1^ (IC_50_) (Figure 2). The positive effect of Andean blueberry in this assay was compared with that of the synthetic antioxidant butylated hydroxytoluene (BHT) at 0.1 mg mL^−1^ (control), showing the dose of 1 mg mL^−1^ having a similar % of inhibition of the lipid peroxidation compared with the control. Thus, the results suggest the potential use of Andean blueberry extracts as natural antioxidant for improving the shelf life of food products.

On the other hand, lipid peroxidation is a major contributor to the loss of cell function involved in many human pathological consequences, such as hepatotoxicity and hepatocarcinogenesis [19], and the zebrafish embryo model has been described to possess homologous oxidative pathways to humans [47]. There is also evidence of the absorption and metabolism of phenolic compounds by the chorion membrane of zebrafish larvae, evidencing this model as valuable for the assessment of healthy biological effects of bioactive compounds [48].

### 3.6. Determination of Antinutritional Lectins

The most known plant components with agglutination properties are the varied lectin proteins, which are able to reversibly bind sugar structures in the blood cells [48]. These proteins are few of the well-known antinutrients in plants and can be found in legumes, seed extracts, fungi, and some fruits; however, their presence in berries has been little studied. Lectins may exert different responses in the human body, from allergies and gastrointestinal problems to bioactive effects related to their selectivity to bind carbohydrate residues of glycoproteins as markers in cancer research [49]. Thus, with this assay, the hemagglutination effect of Andean blueberry extract due to the presence of lectins and other compounds was evaluated using five different concentrations of the extract (2.5, 1.25, 0.625, 0.312, and 0.156 mg mL^−1^). Results showed no agglutination effect of the extracts (data not shown), revealing the possible lack of the lectin, and therefore the absence of an antinutritional effect, in Andean blueberry fruit.

### 3.7. Antimicrobial Activity

*Vaccinium* spp., such as cranberry, blueberry, and bilberry, have shown bactericidal activity against *S. aureus* and *E. coli*, especially in the prevention of urinary tract infections [49]. This activity was associated with the presence of (poly) phenols, mainly flavonol glycosides, anthocyanins, proanthocyanidins, and flavan-3-ols. In this work, only the highest concentration of Andean blueberry aqueous extract tested in this experiment (10 mg mL^−1^) exhibited significant antimicrobial effects toward *S. aureus* and *E. coli*, the percentages of bacterial growth inhibition being 30% and 43%, respectively (data not shown). These results agree with those of previous works showing antibacterial activities in the range 25–100 mg mL^−1^ of blueberry, the inhibitory effect being higher for Gram-positive bacteria than for Gram-negative bacteria [50]. The concentration of 10 mg mL^−1^ of Andean blueberry extract used in the experiment suggests a weak antibacterial effect of this fruit.

### 3.8. In Vitro Gastrointestinal Digestion

The assessment of total phenolic compound content (TPC) and antioxidant capacity during in vitro gastrointestinal digestion of Andean blueberry allowed us to determine how the digestion process affected the stability and, therefore, bioaccessibility, of the dietary (poly) phenols present in this fruit. This experiment resembles the antioxidant role of these fruits in our gastrointestinal tract, where they may exert important beneficial effects against different pro-oxidants (such as diet components) that have been observed to increase oxidative stress before they are absorbed. Our results showed the presence of phenolic contents in all phases of the in vitro digestion (Table 5), representing the availability of these compounds for absorption in the intestinal epithelium and metabolism. No significant changes in TPC were found during the oral and gastric phases, obtaining a bioaccessibility around 85%–90%. Afterward, TPC was recovered in lower contents (51%–56% bioaccessibility), maybe due to degradation processes of these compounds with the intestinal juice treatment, as they are converted to aglycones and glucuronides in the colon. These results agree with those of other authors who found similar losses of (poly) phenols in the intestinal steps [51,52]. Among phenolic compounds, anthocyanins have been found to experience higher losses during gastrointestinal digestion than flavonols and caffeic acid derivatives, all of them being affected by enzymes, pH levels, and secretions in the digestive tract in real physiological conditions [53]. Once the release of phenolic compounds from their matrix into the intestinal lumen has been studied, the further step would be the study of their transport through the epithelium into the body and their availability to be metabolized and absorbed after reaching the colon.

Regarding the antioxidant capacity evaluated by the ABTS method, it showed a high decrease in the gastric phase (Table 5), maybe due to the lower chemical reaction of bioactive compounds with acid pH. Afterward, the antioxidant capacity found during the intestinal and final steps of the gastrointestinal digestion (64–69 µmol TE g^−1^) was significantly higher compared with that found during the initial and oral steps (41–42 µmol TE g^−1^). This fact could be explained by changes in the structural form of (poly) phenols in the intestine, affected by neutral pH and enzymatic activities, which promote multiple forms of metabolites in the intestinal lumen, such as phenolic acids, resulting in a higher ability to scavenge free radicals [53,54]. Finding phenolic compounds after intestinal digestion showed their availability to be metabolized and absorbed after reaching the colon. Apart from showing antioxidant capacity, (poly) phenols may act as digestive enzyme inhibitors, affecting the activity of α-glucosidase, α-amylase, and lipase, which may contribute to the control of diabetes type II and obesity, delivering other health benefits attributed to the ingestion of berries as part of the diet [55,56].

## 4. Conclusions

Andean blueberry is a relevant source of phenolic compounds, mainly anthocyanins, which may be responsible for its high antioxidant capacity. In addition, the freeze-dried extract of Andean blueberry did not show toxicity and could be included in the safe category as a natural ingredient. These characteristics make Andean blueberry suitable to be used as a functional ingredient with potential technological applications in the food industry, such as natural antioxidant or dye, or in the pharmaceutical industry for the development of nutraceuticals. Due to the substantial differences in phytochemical profile among *Vaccinium* spp. and varieties reported in the literature, the identification and quantification of bioactive compounds of Andean blueberry performed in this work is part of the study of this berry as an interesting candidate for the further evaluation of its health benefits through in vivo assays and clinical trials. In this work, the in vitro simulated digestion showed a gradual release of phenolic compounds but a sustained antioxidant activity, increasing the reliability of antioxidant data described for berries. It should be note that further in vivo and clinical studies with Andean blueberry should highlight the real effect of these bioactive compounds in the body, as the absorption and bioavailability could be affected by different interindividual factors.

## Figures and Tables

**Table 1:** Physicochemical characterization of Andean blueberry (*Vaccinium floribundum* Kunth).

Parameters	Content
Weight (g unit ^−1^)	3.5 ± 0.05^1^
Length (cm unit^−1^)	1.75 ± 0.04
Diameter (mm unit^−1^)	8.5 ± 0.75
pH	2.61 ± 0.05
Moisture (%)	88.69 ± 0.08
°Brix	11.17 ± 0.03
Titratable acidity (% citric acid)	1.62 ± 0.00

^1^ Mean values of three determinations ± standard deviation (SD). Fresh weight basis.

**Table 2:** Phenolic compounds detected and characterized tentatively in Andean blueberry samples (*n* = 3) by HPLC-DAD-ESI/MS^n^. Compounds were numbered by their elution order.

Peak Number	Rt (min)	DAD λ (nm)	[M−H]^−^	Fragment Ions (MS^n^)	Phenolic Compounds ^1^
1	6.0	330	707 (2[M−H]^−^) 353	191, 179	3-*O*-Caffeoylquinic acid *
2	10.8	330	353	191	5-*O*-Caffeoylquinic acid
3	16.7	280, 520	465	303	Delphinidin-3-*O*-hexoside I
4	18.5	280, 520	465	303	Delphinidin-3-*O*-hexoside II
5	19.6	280, 520	449	287	Cyanidin-3-*O*-hexoside I
6	20.8	280, 520	435	303	Delphinidin-3-*O*-pentoside
7	21.8	280, 520	449	287	Cyanidin-3-*O*-hexoside II
8	23.9	280, 520	419	287	Cyanidin-3-*O*-pentoside
9	26.6	320	335	179, 161, 131	Caffeoylshikimic acid
10	28.7	360	433	323, 179, 161	Caffeic acid derivative
11	33.6	360	463	301	Quercetin-3-*O*-hexoside I
12	35.2	360	463	301	Quercetin-3-*O*-hexoside II
13	37.5	360	433	301	Quercetin-3-*O*-pentoside I
14	39.6	360	433	301	Quercetin-3-*O*-pentoside II
15	41.2	360	433	301	Quercetin-3-*O*-pentoside III
16	42.8	360	447	301	Quercetin-3-*O*-rhamnoside

^1^ Identification of phenolic compounds based on the ion [M − H]^−^ (m/z) in the negative mode, fragment ion (MS^n^) data, and retention time compared with standards and reference sources. * Dimeric adduct. Rt: retention time.

**Table 3:** Phenolic compounds and carotenoids quantified in Andean blueberry (*n* = 3).

	Concentration	
Phenolic Compounds	(µg/g DW)	
*Hydroxycinnamic acids*		
3-*O*-Caffeoylquinic acid	236.1	± 37.7 ^1^
5-*O*-Caffeoylquinic acid	845.5	± 1.25
Caffeoylshikimic acid	35.8	± 1.58
Caffeic acid derivative	273.0	± 40.0
Total	1390.3	± 78.9
*Anthocyanins*		
Delphinidin-3-*O*-hexoside I	395.7	± 58.5
Delphinidin-3-*O*-hexoside II	274.0	± 50.0
Cyanidin-3-*O*-hexoside I	1963.9	± 140
Delphinidin-3-*O*-pentoside	392.1	± 29.5
Cyanidin-3-*O*-hexoside II	71.1	± 22.3
Cyanidin-3-*O*-pentoside	2289.8	± 327
Total	5386.4	± 567
*Flavonols*		
Quercetin-3-*O*-hexoside I	849.7	± 25.9
Quercetin-3-*O*-hexoside II	70.0	± 13.9
Quercetin-3-*O*-pentoside I	186.0	± 23.1
Quercetin-3-*O*-pentoside II	45.4	± 2.47
Quercetin-3-*O*-pentoside III	683.5	± 23.5
Quercetin-3-*O*-rhamnoside	219.0	± 25.9
Total	2095.5	± 184
Total phenolic compounds	8875.3	± 787
**Carotenoids**		
Lutein	5.94	± 1.34

^1^ Mean values ± standard deviation (*n* = 3). Hydroxycinnamic acids were quantified as 5-*O*-caffeoylquinic acid equivalents, anthocyanins as cyanidin-3-*O*-glucoside equivalents, and flavonols as rutin equivalents.

**Table 4:** Antioxidant capacity of Andean blueberry (*n* = 3).

	Antioxidant Capacity (µmol Trolox g^−1^ DW)		
	ABTS^·^	DPPH^−^	ORAC
Andean blueberry	278.2 ± 59 ^1^	85.1 ± 27	402.2 ± 17

^1^ Mean values ± standard deviation (*n* = 3).

**Table 5:** Total phenolic compounds (TPC), bioaccessibility, and antioxidant capacity determined in the initial, oral, gastric, intestinal, and final phases during in vitro digestion.

Gastrointestinal (GI) Phase	Total Phenolic Content (mg GAE g^−1^)	% Loss	% Bioaccessibility	Antioxidant Capacity (µmol Trolox g^−1^)
Initial	11.27 ± 0.20 ^1^ a			40.53 ± 2.40 b
Oral	10.10 ± 0.94 a	10	90	41.67 ± 1.73 b
Gastric	9.54 ± 1.09 a	15	85	25.94 ± 2.14 c
Intestinal	6.36 ± 0.36 b	43	56	68.67 ± 2.98 a
Final	5.74 ± 0.62 b	49	51	63.97 ± 4.79 a

^1^ Mean values (*n* = 3). Different lowercase letters indicate statistically significant differences among gastrointestinal phases.
